# A family-tailored early motor intervention (EMI-Heart) for infants with complex congenital heart disease: study protocol for a feasibility RCT

**DOI:** 10.1186/s40814-022-01220-y

**Published:** 2022-12-23

**Authors:** Elena Mitteregger, Tineke Dirks, Manuela Theiler, Oliver Kretschmar, Beatrice Latal

**Affiliations:** 1 Child Development Center, University Children’s Hospital Zurich, Zurich, Switzerland; 2 Children’s Research Center, University Children’s Hospital Zurich, Zurich, Switzerland; 3 University of Zurich, Zurich, Switzerland; 4 Paediatric Physiotherapy, Groningen, Netherlands; 5 Swiss Parents’ Association for the Child With Heart Disease (Elternvereinigung Für das Herzkranke Kind), Aarau, Switzerland; 6 Department of Pediatric Cardiology, University Children’s Hospital Zurich, Zurich, Switzerland

**Keywords:** Congenital heart disease, Open-heart surgery, Early motor intervention, Neuromotor development, Physiotherapy, Family-tailored intervention, Parental and child health-related quality of life, Family well-being

## Abstract

**Background:**

Children with congenital heart disease (CHD) undergoing open-heart surgery are at risk for developmental impairments with motor delay manifesting first and contributing to parental concerns. Only a few interventional studies aim to improve neuromotor development in infants with CHD with inconclusive results. We thus developed a family-tailored early motor intervention (EMI-Heart), which aims to promote motor development and family well-being in the first year of life after open-heart surgery.

The primary aim described in this protocol is to evaluate feasibility of EMI-Heart. The secondary aim is to describe the difference between the intervention and control group in motor outcomes and family well-being at baseline, post-treatment, and follow-up.

**Methods:**

This prospective, parallel single-center feasibility randomized controlled trial (RCT) will compare EMI-Heart with standard of care in infants with complex CHD. Sixteen infants and their families, randomly allocated to EMI-Heart or the control group, will participate within the first 5 months of life. Infants assigned to EMI-Heart will receive early motor intervention for 3 months. The intervention’s key is to promote infants’ postural control to enhance motor development and partnering with parents to encourage family well-being.

Feasibility outcomes will be (a) clinical recruitment rate and percentage of families completing EMI-Heart, (b) average duration and number of sessions, and (c) acceptability of EMI-Heart using a parental questionnaire post-treatment, and descriptive acceptability of EMI-Heart to the pediatric physiotherapist.

Secondary outcomes of the intervention and control group will be infants’ motor outcomes and questionnaires assessing family well-being at 3–5 months (baseline), at 6–8 months (post-treatment), and at 12 months of age (follow-up).

We will evaluate feasibility using descriptive statistics. Non-parametric statistical analysis of secondary outcomes will assess differences between the groups at baseline, post-treatment, and follow-up.

**Discussion:**

This feasibility RCT will provide information about a newly developed family-tailored early motor intervention in infants with complex CHD. The RCT design will provide a foundation for a future large-scale interventional trial for infants with CHD after open-heart surgery.

**Trial registration:**

This study protocol (version 1.3, 01.02.2022) was approved by the Cantonal Ethics Commission Zurich (BASEC-Nr. 2019–01,787) and is registered by Clinicaltrials.gov (NCTT04666857).

**Supplementary Information:**

The online version contains supplementary material available at 10.1186/s40814-022-01220-y.

## Background

Congenital heart disease (CHD) is one of the most common birth defects, with 8 of 1000 live-born children being affected world-wide [1, 2]. Achievements in prenatal diagnoses and medical care have increased the survival rate of even complex forms of CHD but have also exposed affected infants to a heightened risk of brain injury and developmental disorders [3]. Additional factors related to complex CHD such as abnormal brain development and perioperative white matter injuries contribute to subsequent neurodevelopmental impairments. These neurodevelopmental impairments comprise motor, cognitive, and sensory outcomes [4].

Infants born with complex CHD are at risk for a variety of developmental impairments. Motor development is the first domain in which impairment becomes apparent in the first year of life with a prevalence of 40–60%. Other developmental impairments, such as language disorders and behavioral and learning difficulties, may occur throughout childhood but often only become evident later at school age [5]. Evidence shows that early motor developmental abnormalities persist into childhood, adolescence, and adulthood [6].

Despite strong evidence of motor development impairments in infants with complex CHD, no targeted, specific, or tailored treatment is available. However, there is a clear need for an early motor intervention that aims to prevent problems before they manifest or mitigate existing ones and reduce difficulties later in life [7].

### Postural control and sitting

In the first few months of life, typically developing infants spend most of the time they are awake supine, held, or in supported sitting. Time in sitting increases with the ability to learn sitting freely. Motor experiences such as sitting have cumulative consequences, a cascade of effects in other developmental domains such as cognition, social, and language development [8]. Infants’ points of view change in sitting from supine. Infants learn how to control their own body against gravity with hands free to explore toys and their surroundings. They interact variously with objects, people, and their environments [9, 10]. Postural control in sitting also enables the interaction promoting face-to-face exchange and joint attention with their caregivers [11].

Two theories describe the importance of postural control in early motor development. One theory describes postural control as a complex and dynamic process of learning and adapting to diverse environmental forces and tasks [12, 13]. The other theory describes postural control as an innate, genetically determined aspect of behavior that changes with exploration in infancy [14]. Both theories agree that postural control is variable, is affected by many factors, and is a key element in early motor development, including learning to sit independently.

In infants at risk for neuromotor disorders, such as infants with CHD, postural control is reduced. Compared to typically developing infants, they spend more time supine and only start sitting later. Thus, the opportunity to actively explore their body and surroundings is reduced from early infancy. Studies have shown that improvement of postural control in infants at high risk for developmental delay in sitting facilitates their motor and cognitive development [15–18].

Kretch et al. [11] demonstrated that caregivers were most likely to provide learning opportunities when infants were sitting. Their findings suggest that early intervention should focus on improving postural control. Infants with neuromotor delay should be positioned early in supported sitting in a way that allows face-to-face contact with the parents before they can sit independently. This strategy opens new motor and cognitive learning opportunities. Motor learning is an essential part of early motor intervention programs for infants at risk for developmental disorders.

### Early intervention

A wide body of literature underlines the importance of early intervention for infants at high risk for neurodevelopmental impairments such as cerebral palsy [15, 19–21]. The World Health Organization even states that it is crucial to identify infants at risk for neurodevelopmental disorders, establish a close relationship between parents and health care professionals, and provide early intervention [22]. Infants with CHD that undergo open-heart surgery together constitute such a population at risk for motor developmental delay.

However, motor interventions that focus on this patient group are sparse. Few interventional studies exist that start in infancy and aim to improve motor development. Cohort studies [23–25] and single case studies [26, 27] have investigated the influence of early physiotherapy for infants with CHD. Although the results are inconclusive due to their low level of evidence and heterogeneity, early motor interventions seem to positively influence motor development in infants with CHD.

A considerable proportion of infants with CHD are unable to tolerate the prone position. This might be due to surgery, lack of prone positioning, discomfort, or parental protection. Dagenais et al. [28] investigated the prone performance in infants after open-heart surgery and concluded that better scores in prone performance of the Alberta Infant Motor Scale [29] predicts earlier onset of walking. Uzark et al. [30] found that infants after open-heart surgery that performed the prone position daily had significantly better motor skills than those who did not. Thus, these studies emphasize the importance of promoting the prone position for infants with CHD.

### Parental engagement

Parents offer their children many learning opportunities during their upbringing. Motor delay contributes to parental concerns and difficulties in child–parent wellbeing. In our qualitative study about the experience of parents of infants with CHD, parents reported that they were reluctant to challenge their infants [31]. They feared over-exerting them and watched them constantly. Parental overprotection, which occurs more frequently with infants with CHD, might negatively influence children’s motor development. Reduced physical activity that starts early in life most likely continues. This assumption implies that early support of parents is equally important as early motor support of their infants. Parental attendance and active engagement play a key role in early intervention [32–34].

Depending on the severity of children’s heart disease, parents’ resources, and family support, stress can last beyond children’s hospital stays [35]. This impacts parent–child relationships because parental well-being is crucial for children’s health and adjustment. Our study about the experience parents of children with CHD [31] underpins the importance of involving parents as experts in their children and as partners in decision-making about their care. Parents appreciate medical information that helps them to better understand and support their children’s development and thus provide the best possible outcome for children and their families.

An effective intervention that can prevent maladaptive plasticity of infants’ brains has to (a) start early, (b) occur at high frequency and require the child to be active (c) be playful and goal directed, (d) be tailored to each family separately, and (e) engage caregivers as equal partners [36–38].

There is a lack of an early motor intervention specifically tailored to infants with CHD and their families. Thus, the purpose of the study described in this protocol is to test the feasibility of an early newly developed family-tailored early motor intervention in infants with complex CHD (EMI-Heart) after open-heart surgery. There is equipoise between the intervention and control group therefore we chose a RCT design. The results of this feasibility RCT will lay the foundation for a larger RCT to test the efficacy of this intervention.

## Methods/design

### Aim

The primary aim of this study is to evaluate feasibility using measures like recruitment and adherence rate, parental acceptability of EMI-Heart, and acceptability to the pediatric physiotherapist providing EMI-Heart. The secondary aim is to describe the difference between the intervention and control group in motor outcomes and measures of family well-being. The results of this feasibility intervention trial will provide the foundation for a larger future RCT.

### Study design

This feasibility study is a prospective single-center single-blinded, two-arm parallel RCT that compares EMI-Heart to standard of care for infants with CHD after open-heart surgery.

**Fig. 1:**
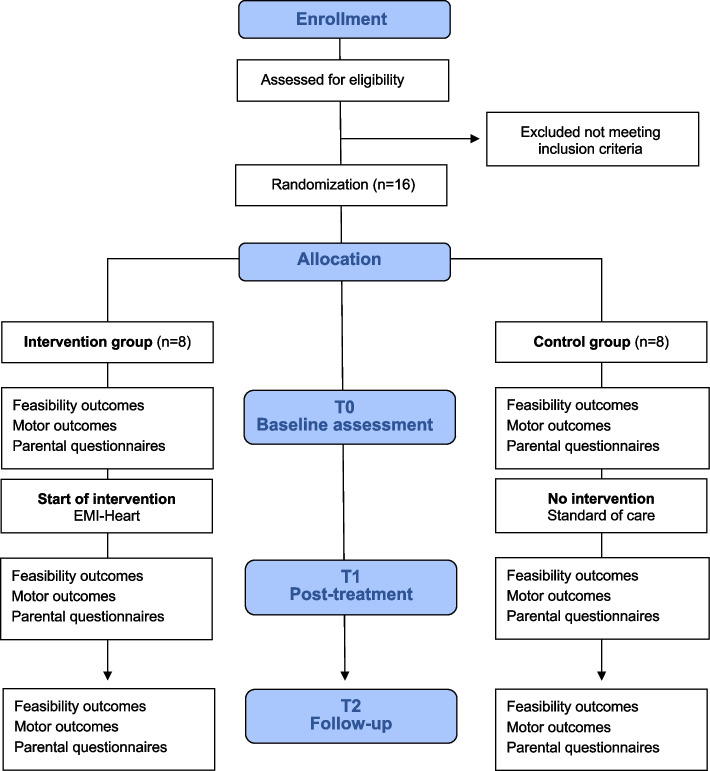
Flow diagram of the study procedure according to Consort 2010. T0: baseline; T1: post-treatment; T2: follow-up

**Fig. 2:**
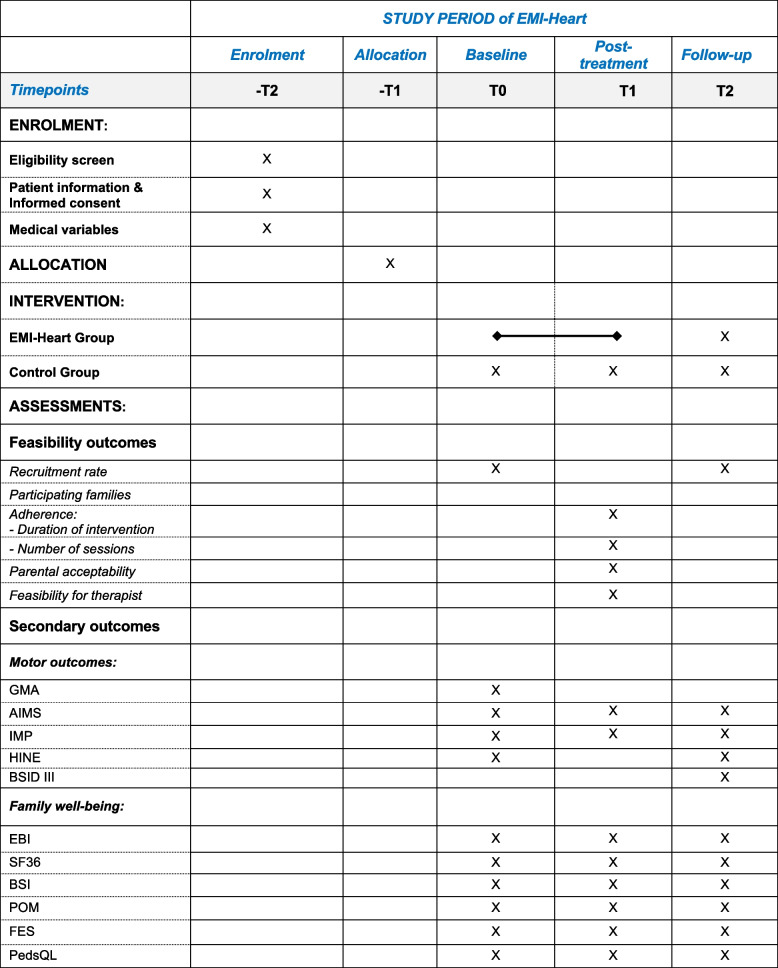
Schedule of enrolment, interventions, and assessments according to SPIRIT 2013. IMP (Infant Motor Profile), AIMS (Alberta Infant Motor Scale), GMA (General Movements Assessment), HINE (Hammersmith Infant Neurological Examination), BSID III (Bayley Scales of Infant and Toddler Development), EBI (Eltern Belastungs Inventar), SF36 (Quality of life Short Form 36), BSI (Brief Symptom Inventory), POM (Parental Overprotection Measure), FES (Family Empowerment Scale), PedsQL (Pediatric Quality of Life Inventory)

Sixteen participants meeting the eligibility criteria will be recruited over a period of 12 months. Infants will be randomized and assigned to the intervention (*n* = 8), or control group (*n* = 8) determined by a computer-generated random sequence. This will ensure that baseline characteristics of each group will be as similar as possible. Groups’ allocation will be concealed. Sealed opaque and numbered envelopes will be sequentially opened to preserve concealment by members of the Children’s Hospital that are not involved in this project. Both the intervention group and the control group will be assessed at baseline (T0), at post-treatment (T1) after nine sessions approximately 3 months later, and at follow-up (T2) at 12 months of age (see Figs. 1 and 2). All assessments will be video recorded. Five assessors (CE, MS, RE, BL, RC) blinded to group allocation and not involved in the intervention will score the video recordings. Assessments will be scored independently, and results will not be shared between assessors. Parents and the pediatric physiotherapist (PT) providing the intervention will not be blinded to the intervention.

### Study participants and recruitment

All study participants will be assessed for study inclusion and recruited at the University Children’s Hospital Zurich consecutively by members of the Department of Cardiology and the investigator EM before baseline. We will include participants into the trial with the following criteria: (1) infants with CHD (e.g., tetralogy of Fallot, pulmonary atresia, transposition of the great arteries, atrial/ventricular septal defects, double outlet right ventricle), (2) infants born ≥ 37 weeks gestational age, (3) infants aged 3–5 months who underwent open heart surgery with cardiopulmonary bypass once within the first 5 months of life, (4) infants discharged home before the age of 6 months, (5) informed consent of infants’ parents documented by signature, and (6) families living within an hour’s journey from the Children’s Hospital.

We will exclude (1) infants with univentricular heart defects like hypoplastic left-heart syndrome, because they need to undergo several planned open-heart surgeries within the first year of life; (2) infants with syndromes that are often associated with CHD ﻿and worse neurodevelopmental outcomes such as trisomy 21, 22q11 microdeletion, CHARGE, Noonan, and the VACTERL association; and (3) large cerebral and clinically manifest lesions, (4) infants whose parents have an inadequate understanding of the German language and are thus unable to comprehend the patient information.

### Patient and public involvement

The Swiss parents’ association for the child with heart disease (Elternvereinigung für das herzkranke Kind) will provide us with advice for the conduct of this study and for the recruitment of families. MT, a parental stakeholder, is co-author of this manuscript. In the development of EMI-Heart, we performed interviews with parents of children with CHD who underwent open-heart surgery within the first 6 months of life. The result of this qualitative study describes a variety of burdens and needs parents had experienced and which determines the design of EMI-Heart [31].

All families will be informed of the burden of the intervention and given the option to stop at any stage. All eligible families completing the study will receive an individual report of the results and a general report of study results when data analyses are completed.

### Study groups

#### Intervention group: EMI-Heart

Infants randomly assigned to the intervention group receive EMI-Heart. The investigator EM, a senior pediatric PT, specialized in early motor development will provide all interventions to maximize fidelity of EMI-Heart. The second author TD, a senior pediatric PT with extensive knowledge in early intervention, will support the quality of the content of EMI-Heart and its implementation in practice by discussing video recordings of the intervention with EM. To improve adherence to the intervention EM regularly contacts parents via telephone, text messaging and emails. EMI-Heart will start after hospital discharge and baseline assessments and will last for approximately 3 months, take place once a week or fortnightly for 45–60 min per session. The intervention will consist of nine treatment sessions: three sessions at home, three at the children's hospital, and three online, ideally in an alternating order.

EMI-Heart is based on our qualitative study, which describes parental experience of their infants’ neuromotor development after open-heart surgery. Parents wanted to actively support their infants’ development and be respected as experts of their infants. Therefore, the intervention’s key is to promote postural control to enhance motor development in infancy [13, 39, 40], and partnering between parents and the PT at eye-level to encourage family well-being, as recommended in current research on pediatric rehabilitation [32–34]. EMI-Heart is family-tailored to each unique family. It is adapted to infants’ motor abilities and considers family’s own experiences and wishes. EMI-Heart’s key aims are intertwined with each other and addressed as described below:

**Promotion of infants’ postural control**

Infants with CHD after open-heart surgery are often not exposed to the prone or the early sitting position due to perceived discomfort and/or parental protection.

- The PT creates safe and playful postural activities in prone and early sitting and support parents how to stimulate their infants’ postural activities repeatedly and joyfully in daily life activities. Prone and sitting positions are explored and adjusted to infants’ needs. External support, e.g., towels or cushions placed under infants’ armpits and chest can facilitate head lifting in prone. In early sitting parents’ hands and body, furniture, cushions, and toys are used to stimulate trunk/head control, reaching, and grasping activities.
- As soon as infants improve their postural control (e.g., lifting the head with more ease in prone, visible enjoyment, less wobbling of the head, better ability to look around, goal-directed reaching and grasping activities in sitting), PT and parents gradually reduce external support.

**Fig. 3:**
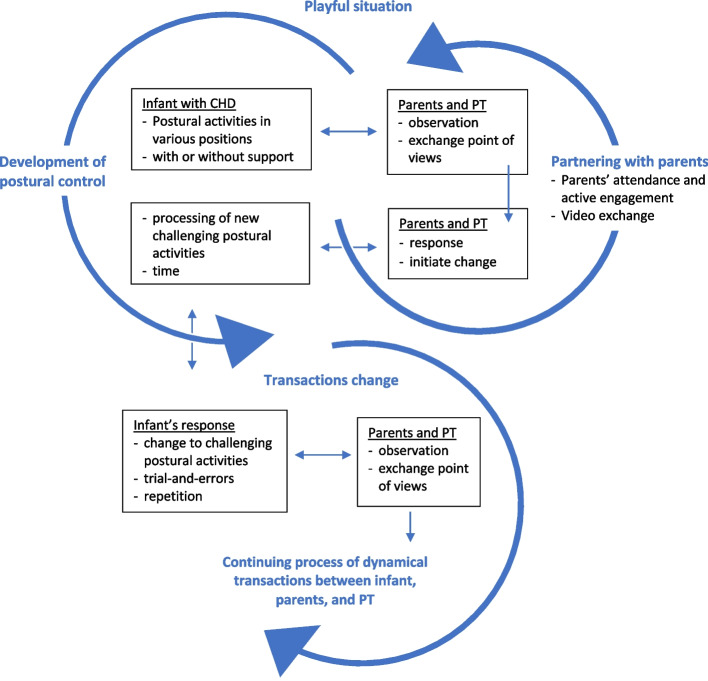
EMI-Heart, Transactional Model of Change. The spiral illustrates the continuing interplay between infant’s postural activities and responses on parents and the PT’s actions and vice versa. CHD: congenital heart disease, PT: physiotherapist

The continuing interplay between infant’s postural activities and responses on parents and the PT’ actions and vice versa are illustrated in Fig. 3. Two dynamic elements are interacting as an ongoing spiral: (1) infant’s postural activities and behavior and (2) partnering between parents and the PT. These elements change constantly due to transactions between the infant, parents, and the PT. These transactions are embedded in daily life activities, which in turn are part of each family’s ecological environment.

**Partnering with parents**

a Parents’ attendance and active engagement
   In each session, parents are present and actively engaged. Parents and the PT act as equal partners, share their responsibility and openly discuss their point of views. Parents are their infants’ experts and share their experience and wishes with the PT. The PT in turn shares her/his professional and empirical knowledge*,* clinical reasoning, and current research evidence in early intervention.
b Encouragement of parents’ confidence and family well-being
   In our qualitative study, parents expressed the need to strengthen their confidence and learn how to trust their children’s abilities [31]. The PT uses different strategies to encourage parents’ confidence and family well-being.
  i *Joint exploration*: parents often feel insecure in handling their infants after open-heart surgery and therefore are reluctant to try out new positions like prone and early sitting. The PT shows parents how they can confidently handle their infants and encourages them to trust their infants’ ability to explore the environment (see “Promotion of infants’ postural control” section). Together the PT and parents jointly explore positions in which both infants and parents feel comfortable.
  ii *Promoting interplay between infants and parents:* the PT promotes face-to-face interaction between parents and infants with verbal encouragement. This stimulates joint play and joyful infant-parent interaction. Early sitting with and without support, e.g., does not only enhance active body control against gravity, but also enables infants to explore toys and interact with their parents and the environment.
  iii *Video-exchange:* all intervention sessions are video-recorded and made available to parents. Additionally, parents are invited to send homemade video-clips of their infants’ daily activities to the PT. Watching those video-recordings enables parents and the PT to see themselves and infants from a distance. This exchange encourages partnering with parents and active engagement. Homemade video-clips enable the PT to see how parents implement EMI-Heart in daily life. Studies showed that video feedback positively promotes parental engagement and increases parental capacity to read and respond to their children’s signals [41, 42].
  iv *Online sessions in addition to visits at home and at the hospital:* Online sessions promote parental engagement as the PT cannot physically interact with the family. Parents show the PT how they stimulate infants’ postural activities in real-life situations. The PT answers parental questions and provides feedback when necessary. This also allows the PT to virtually exchange video-clips of the intervention and demonstrate how well parents are stimulating their infants’ development. Online sessions save travel time, are flexible to plan and are easy to access for both parents and the PT. This is in line with Rosenbaum et al. [43, 44] who described that virtual therapy improved parental skills and understanding of how to support their children.

#### Control group: standard of care

Infants randomly assigned to the control group receive standard of care for infants with CHD after open heart surgery, which includes cardiac surveillance, counseling, and screening at the University Children’s Hospital Zurich and standardized developmental check-ups by their pediatrician. Pediatric PT is not normally part of standard of care. However, some infants may receive physiotherapy if they present with obvious signs of motor developmental delay. Infants receiving physiotherapy are usually treated at pediatric outpatient clinics once a week or fortnightly for approximately 3 months.

### Outcome measures

The feasibility will be measured by (a) clinical recruitment rate; (b) percentage of families completing EMI-Heart, (c) adherence: average duration and number of sessions used in EMI-Heart; and (d) the acceptability of EMI-Heart using a parental questionnaire post-treatment (see Table 1). We developed a parental acceptability questionnaire based on the core elements of EMI-Heart. It consists of 18 questions with a four-point Likert scale response. The feasibility of the EMI-Heart intervention for the providing pediatric physiotherapist will be described (see Table 1).

**Table 1:** Feasibility outcomes

	***Description***
Recruitment rate	Sum of recruitment rate and reasons of withdrawal
Participating families	Percentage of families completing the study
Adherence	
*Duration of the intervention*	Time between T0 to T1 expressed in months, weeks
*Number of intervention sessions*	Number of sessions provided per intervention infant
Parental acceptability	18 items Likert scale 0–4 (0 do not agree–4 totally agree) at timepoint T1 (post-treatment)
Feasibility for pediatric physiotherapist	Description of the feasibility of EMI-Heart to physiotherapist (setting, travel time, preparation, and follow-up)

Secondary outcomes of the intervention and control group will be infants’ motor outcome and family well-being, see Table 2. We will assess infants’ motor development and family well-being at timepoints T0, T1, and T2 in both the intervention and control groups. Infants’ motor outcomes include the Infant Motor Profile [45], the Alberta Infant Motor Scale [29] and the Bayley Scales of Infant and Toddler Development third version [46]. Baseline variables include the General Movements Assessment [47, 48] and the Hammersmith Infant Neurological Examination [49, 50]. These validated and reliable assessments are widely used in practice and research. Validated German versions will be used if available. All assessments will be video recorded and evaluated by assessors blinded to group allocation. Parental questionnaires will evaluate parents’ and infants’ health-related quality of life, parental mental health and stress experience, infants’ protection, and parental empowerment. The questionnaires will be provided as a survey to be completed online with Research Electronic Data Capture (REDCap) electronic data capture tools hosted at the University Children’s Hospital Zurich [51, 52]. Medical and cardiac valuables will be derived from the electronic medical charts of the hospital’s data management system.

**Table 2:** Secondary outcomes

		**Timepoints**		
		**T0**	**T1**	**T2**
***Motor assessments***				
Infant Motor Profile [45]		x	x	x
Alberta Infant Motor Scale [29]		x	x	x
General Movement Assessment [47]		x		
Hammersmith Infant Neurological Examination [49]		x		x
Motor domains of the Bayley Scales of Infant and Toddler Development III [46]				x
***Parental questionnaires assessing family well-being***				
Infants’ quality of life	Pediatric Quality of Life Inventory [53]	x	x	x
Parents’ quality of life	Short Form Survey-SF 36 [54, 55]	x	x	x
Parental mental health	Brief Symptom Inventory 18 [56]	x	x	x
Parental stress experience	Parental Stress Index [57]	x	x	x
Infants’ protection	Parental Overprotection Measure [58]	x	x	x
Parental empowerment	Family Empowerment Scale [59]	x	x	x

### Sample size and data analysis

Based on previous clinical data of the University Children’s Hospital [60] of the last 3 years and on previous intervention studies [61] approximately 30% of infants undergoing open-heart surgery within the first 5 months of life will meet our inclusion criteria. This corresponds to a sample size of approximately 16 infants who we aim to recruit for our feasibility study. This study will not be powered for statistical hypothesis testing. We are aware that our results will not be generalizable to a wider population. Nonetheless, we decided to keep to this sample size as the Covid pandemic complicates recruitment. Open-heart surgeries are being canceled or postponed leading to the exclusion of initially eligible infants. The study described in this protocol aims to determine the intervention’s feasibility of conducting a larger future multi-center RCT to test the efficacy of this intervention. This study trial will be conducted according to the SPIRIT [62] and the TIDieR statement [63], and reported according to the CONSORT statement [64].

Clinical data will be analyzed with statistics R or the Statistical Package for Social Sciences. Descriptive statistics of the feasibility outcomes will be calculated, including means and SD or medians and IQRs for continuous variables and/or for categorical variables. Recruitment will be measured by summarizing recruitment rate and reasons of withdrawal compared to available patients listed in the screening log. Secondary outcomes will be collected at each timepoint and summarized descriptively for the intervention and the control group. Secondary outcomes will assess differences between the intervention and control group at baseline, post-treatment and follow-up using an intention-to-treat analysis. Non-parametric methods will be used if parametric assumptions are violated.

## Discussion

The protocol of this feasibility RCT will provide information about a newly developed family-tailored early motor intervention in infants with complex CHD after open-heart surgery. To the best of our knowledge, no early motor intervention exists that specifically focuses on infants with CHD, aiming to promote infants’ postural control and family well-being. This study will provide preliminary results of the feasibility of EMI-Heart and used outcome measures. Additionally, this study will provide information on the feasibility of home visits and online treatment sessions that will allow interventions to be adjusted to the real-life situations of families. In a future step, we plan to analyze all video recordings of the intervention sessions using a qualitative content analysis. Identifying the transactions between parents, infants, and the therapist will enable the reproducibility of this newly developed intervention.

## Supplementary Information

**Additional file 1.** Ethical approval.

**Additional file 2.** Parent information and informed consent.

**Additional file 3.** Study specific monitoring plan.

**Additional file 4.** Trial Register Data Set.

**Additional file 5.** Spirit Checklist.

**Additional file 6.** Consort Checklist.

**Additional file 7.** TIDieR Checklist.

## Data Availability

The datasets that will be used and analyzed will be available from the corresponding author on reasonable request.

## Abbreviations

CHD: Congenital heart disease
EM: Elena Mitteregger
TD: Tineke Dirks
MT: Manuela Theiler
BL: Beatrice Latal
OK: Oliver Kretschmar
CE: Christa Einspieler
MS: Maaike Sprong
RE: Rian Eijsermans
RC: Rachel Cott
EMI-Heart: Early motor intervention for infants with complex congenital heart disease
