# Supplementary material for: A family-tailored early motor intervention (EMI-Heart) for infants with complex congenital heart disease: study protocol for a feasibility RCT
Source: Pilot Feasibility Stud. 2022 Dec 23;8:263. doi: 10.1186/s40814-022-01220-y (PMC9784271; doi:10.1186/s40814-022-01220-y)
Supplement: Supplementary file 1 — Additional file 1. Ethical approval. [file 40814_2022_1220_MOESM1_ESM.pdf]

Kanton Zürich
**Kantonale Ethikkommission**

**Prof. Dr. med. Konrad E. Bloch**
Präsident Abteilung B

**Dr. med. Peter Kleist**
Geschäftsführer
Stampfenbachstrasse 121
Postfach
8090 Zürich
Telefon +41 43 259 79 70
Fax +41 43 259 79 72
admin.kek@kek.zh.ch
www.zh.ch/kek

Universitäts-Kinderspital Zürich
Abteilung Entwicklungspädiatrie
MScPT Elena Mitteregger
Steinwiesstrasse 75
8032 Zürich

15. Februar 2022 / ere

# Verfügung der Kantonalen Ethikkommission Zürich

| | |
|---|---|
| **Wesentliche Änderung** | |
| **Eingereicht am** | 03.02.2022 und 04.02.2022 |
| **BASEC-Nr.** | 2019-01787 |
| **Projekttitel** | A family centred early intervention programme to improve motor development of infants with severe congenital heart disease |
| **Zentrum** | MScPT Elena Mitteregger, Universitäts-Kinderspital Zürich |

## Entscheid

**Die Bewilligung wird erteilt.**
Allfällige weitere Bewilligungspflichten sind zu beachten (siehe Hinweise im Anhang).

## Entscheidverfahren

☐ vereinfachtes Verfahren ☒ Präsidialentscheid

Die Ethikkommission bestätigt, dass sie nach ICH-GCP arbeitet.

## Gebühren

Betrag: CHF 150.- Tarifcode: 6.0
Gemäss der geltenden Gebührenordnung von swissethics.

**Rechtsmittelbelehrung**
Gegen diesen Beschluss kann innert 30 Tagen, von der Mitteilung an gerechnet, beim Regierungsrat des Kantons Zürich schriftlich Rekurs eingereicht werden. Die Rekursschrift muss einen Antrag und dessen Begründung enthalten. Der angefochtene Entscheid ist beizulegen oder genau zu bezeichnen. Die angerufenen Beweismittel sind genau zu bezeichnen und soweit möglich beizulegen.

**Kopie an**

- ☒ Sponsor
- ☐ Swissmedic
- ☐ Bundesamt für Gesundheit
- ☐ beteiligte Ethikkommission
- ☐ andere:

i.V.

Prof. Dr. med. Konrad E. Bloch
Präsident

Dr. med. Peter Kleist
Geschäftsführer

Anhang: - Liste der eingereichten Dokumente

## Eingereichte Dokumente für das Hauptzentrum

**MScPT Elena Mitteregger, Universitäts Kinderspital Zürich, Zürich**

| Dokument | Dok.Datum | Version |
|---|---|---|
| **1. Cover Letter** | | |
| emi-coverletter-ethik-20-09-2019.pdf | 20/12/2019 | |
| begleitbrief-amendement-emi-heart-16-04-2021.pdf | 16/04/2021 | |
| begleitbrief-amendement-emi-heart-03-02-2022.pdf | 03/02/2022 | |
| **3. Participant information sheet and informed consent (ICF)** | | |
| emi-parent-information-informed-consent-further-research-use-version-1-3-01-02-2-track-change.docx | 03/02/2022 | 1.3 |
| emi-parent-information-informed-consent-further-research-use-version-1-3-01-02-2-clean.docx | 03/02/2022 | 1.3 |
| ses-fragebogen-elterninformation-einversta-ndnis-version-1-0-02-02-2022.pdf | 02/02/2022 | 1.0 |
| **4. Study plan (protocol), signed and dated** | | |
| emi-protocol-version-1-3-01-02-2022-track-change.docx | 03/02/2022 | 1.3 |
| emi-protocol-version-1-3-01-02-2022-clean.docx | 03/02/2022 | 1.3 |
| **4a. Monitoring plan** | | |
| emi-monitoring-plan-version-1-1-16-04-2021-trackchange.pdf | 16/04/2021 | 1.1 |
| fzk-monitoringvereinbarung-emi-heart-studie-version-1-0.pdf | 15/03/2021 | 1.0 |
| emi-monitoring-plan-version-1-1-16-04-2021-clean.pdf | 16/04/2021 | 1.1 |
| **5. CRF (Case Report Form)** | | |
| emi-casereportforms-version1-0.pdf | 16/09/2019 | 1.0 |
| emi-clino-serious-adverse-event-form-version-1-0-07-09-2019.pdf | 07/09/2019 | 1.0 |
| **6. Investigator's CV, dated** | | |
| cv-elena-mitteregger-2019.pdf | 07/09/2019 | |
| **7. Investigator's proof of GCP training** | | |
| gcp-modul-1-2-mitteregger-07-09-2019.pdf | 07/09/2019 | |
| gcp-modul-3-mitteregger-07-09-2019.pdf | 07/09/2019 | |
| cv-latal19-version-1-0.pdf | 20/12/2019 | |
| nachweis-gcp-latal-version1-0.pdf | 20/12/2019 | |

## 10. Insurance

see doc/cat: **4**, page/ref: **12**

## 11. Other documents handed over to study participants

No other documents handed over to study participants

## 12. Details on nature and scope/value of compensation for participants

There is no compensation for the participation in this study

## 14. Information on secure handling of biological material and personal data, and in particular on the storage thereof

see doc/cat: **7**, page/ref: **13**

## 39. Miscellaneous / Varia

| | | |
|---|---|---|
| emi-tests-and-questionaires-version-1-3-01-02-2022-track-change.docx | 01/02/2022 | 1.3 |
| emi-feasibility-version-1-0-01-02-22.pdf | 01/02/2022 | 1.0 |
| emi-tests-and-questionaires-version-1-3-01-02-2022-clean.docx | 01/02/2022 | 1.3 |
| ses-redcap-fragebogen-version-1-0.pdf | 04/02/2022 | 1.0 |

UNIVERSITÄTS-
KINDERSPITAL
ZÜRICH

*Forschungszentrum*
*für das Kind FZK*

Kantonale Ethikkommission Zürich
Stampfenbachstrasse 121
8090 Zürich

**Universitäts-Kinderspital Zürich – Eleonorenstiftung**
Prof. Dr. med Bea Latal, MPH
Abteilung für Entwicklungspädiatrie
Email: bea.latal@kispi.uzh.ch
Tel: +41(0)44 266 7751

Elena Mitteregger, MScPT
Email: elena.mitteregger@kispi.uzh.ch
Tel: +41(0)44 266 7690

Kinderspital Zürich
Universitäts-Kinderkliniken
Steinwiesstrasse 75
8032 Zürich
Schweiz

Zürich, 03.02.2022

***A family centred early intervention programme to improve motor development of infants with severe congenital heart disease*** (BASEC-Nr. **2019-01787)**

Sehr geehrte Damen und Herren

Hiermit sende ich Ihnen eine Liste der beabsichtigten Ergänzungen zum oben genannten «übrigen klinischen Versuch» gemäss Kapitel 4 KlinV.

**Art und Grund der Einreichung:**
(Non-substantial and substantial) Amendement

**Bezug zu bereits eingereichten Unterlagen:**
Dieses Amendement bezieht sich auf den am 07.01.2020 bewilligten «übrigen klinischen Versuch» gemäss Kapitel 4 KlinV mit der BASEC-Nr. **2019-01787**«Family centred intervention for infants with CHD» sowie dessen bewilligten Amendments von 27.04.2021.

**Rechnungsadresse:**

**Prof. Dr. med. Bea Latal MPH**
Leiterin Abteilung Entwicklungspädiatrie
Universitäts-
Kinderspital Zürich
Abteilung Entwicklungspädiatrie,
Kreditorenflow
Elena Mitteregger, MScPT
Steinwiesstrasse 75
8032 Zürich

UNIVERSITÄTS-
KINDERSPITAL
ZÜRICH

*Forschungszentrum
für das Kind FZK*

**Anmerkungen zum Amendement:**

Wir erkannten bei Studienbeginn, dass es einfacher ist die Assessments der Kinder im Kinderspital durchzuführen, um ein standardisiertes Testen zu ermöglichen. Es gibt Eltern, die an unserer Elternbefragung teilnehmen wollten, aber nicht aktiv an der Studie teilnehmen möchten. Um eine familienzentrierte Forschung zu gewährleisten, ist zudem die Meinung der Teilnehmenden bedeutsam.

Vor diesem Hintergrund möchten wir die folgenden Neuerungen einreichen:

Die Durchführung der Assessments wurde ins Kinderspital verlegt, da dann für alle Kinder die gleichen Bedingungen gelten und der Ablauf standardisiert werden kann. Es zeigt sich auch, dass dies für Eltern einfacher zu organisieren ist, da sie Termine am Kinderspital kombinieren können. Die Reihenfolge der Interventionstherapien findet in Absprache mit den Eltern abwechselnd im Kinderspital, zu Hause und online mittels Videokonferenzen statt (siehe Protocol, Seite 9, Punkt 2.4 unter b) Intervention Group).

Es gab Eltern, die nicht an unserer Interventionsstudie teilnehmen wollen, sich aber bereit erklären Angaben zu ihrer Lebenssituation zu machen. Das hilft uns Faktoren zu erheben warum Eltern an unserer Interventionsstudie teilnehmen oder nicht.

Da diese Pilotstudie eine Feasibilitystudie ist und als Vorbereitung für eine gross angelegte Studie dient, haben wir beschlossen einen Feasibility Fragebogen zu erstellen und den Eltern der Interventionsgruppe nach Ende der Intervention zuzusenden. Das dient dazu ein differenziertes Feedback von Seiten der Eltern zu erhalten.

- siehe Feasibility Questionnaire: EMI_Feasibility_version 1.0 (01.02.22)

Um die Inhalte der Intervention bezüglich der Interaktionen zwischen Therapeutin, Eltern und dem Kind analysieren zu können, werden zudem alle Therapiestunden auf Videos aufgezeichnet. Die Auswertung soll mittels einer qualitativen Analyse zu einem späteren Zeitpunkt erfolgen.

**Liste der veränderten Dokumente:**

- EMI_Protocol_version 1.3 (01.02.2022)
- EMI_Parent Information, Informed Consent, Further Research Use_version 1.3 (01.02.2022)
- EMI_Tests and Questionaires_version 1.3 (01.02.2022)
- EMI_Feasibility_version 1.0 (01.02.22)
- SES Fragebogen_ Elterninformation_Einverständnis_version 1.0. (02.02.2022)

Die vorgenommenen Änderungen sind markiert. Anfügen möchten wir, dass durch das Amendement die Testung standardisiert und vereinfach wird und die Erhebung der Lebenssituation sowie das Feedback der Eltern uns erlaubt Hintergründe an der Studienteilnahme besser zu verstehen. Alle Änderungen verändern den Studienablauf (zeitlich sowie inhaltlich) nicht grundlegend. Bei Fragen oder Unklarheiten stehen wir Ihnen gerne zur Verfügung.

Für die wohlwollende Prüfung der Dokumente danken wir Ihnen bereits im Voraus.

Freundliche Grüsse,

*Prof. Dr. med. Bea Latal*

*Elena Mitteregger, MScPT*

UNIVERSITÄTS-
KINDERSPITAL
ZÜRICH

*Forschungszentrum für das Kind FZK*

*Sponsor* *Principal Investigator*
