# Supplementary material for: A family-tailored early motor intervention (EMI-Heart) for infants with complex congenital heart disease: study protocol for a feasibility RCT
Source: Pilot Feasibility Stud. 2022 Dec 23;8:263. doi: 10.1186/s40814-022-01220-y (PMC9784271; doi:10.1186/s40814-022-01220-y)
Supplement: Supplementary file 2 — Additional file 2. Parent information and informed consent. [file 40814_2022_1220_MOESM2_ESM.pdf]

Elterninformation

Frühintervention von Säuglingen mit angeborenem Herzfehler

Liebe Eltern
Wir möchten Sie hiermit über unsere klinische Studie informieren. Wir lassen Ihnen daher diese Information zukommen und bitten Sie Ihre Zustimmung für die Teilnahme Ihres Kindes an der Studie zu prüfen. Sie als Eltern können stellvertretend einwilligen.

1. Ziel des Projektes

Das Ziel dieser Studie ist es die Entwicklung von Säuglingen mit angeborenem Herzfehler nach offener Herzoperation im ersten Lebensjahr zu begleiten.
Wir möchten die Standard-Nachsorge dieser Säuglinge, wie sie am Kinderspital üblich ist, mit einem neuen Programm zur Entwicklungsunterstützung vergleichen. Das bedeutet, dass es zwei Gruppen von Studienteilnehmern geben wird. Dadurch erhoffen wir uns mögliche Entwicklungsschwierigkeiten aufdecken zu können und zu verstehen wie Säuglinge nach Herzoperation in ihrer Entwicklung am besten unterstützt werden können. Aus diesem Grund möchten wir Ihr Kind gerne in unsere Studie einschliessen, auch wenn Ihr Kind nicht von einer Entwicklungsverzögerung betroffen ist.

2. Auswahl der Teilnehmenden

Sie werden für diese Studie angefragt, weil Ihr Säugling am Herzen operiert wurde. Bisher gibt es keine spezifische Entwicklungsunterstützung für Säuglinge nach einer Herzoperation. Aus diesem Grund ist es uns ein grosses Anliegen herauszufinden, ob diese für die Entwicklung Ihres Kindes unterstützend sein kann.

3. Allgemeine Informationen zum Projekt

In enger Kooperation zwischen der kardiologischen und entwicklungspädiatrischen Abteilung am Kinderspital Zürich sind Säuglinge mit angeborenem Herzfehler für die Studienteilnahme vorgesehen. Die Studie wird nach geltenden schweizerischen Gesetzen und international anerkannten Grundsätzen durchgeführt und von der kantonalen Ethikkommission Zürich begutachtet und bewilligt.

4. Projektablauf

Bei Studienteilnahme wird Ihr Kind zufällig mit Hilfe eines Computerprogramms entweder in die Kontrollgruppe (Standard-Nachsorge) oder in die Interventionsgruppe (zusätzliche Entwicklungsbegleitung) eingeteilt. Die Chancen in die Interventionsgruppe eingeteilt zu werden stehen (1:1).

Innerhalb dieser Studie wird Ihr Kind unabhängig von der Gruppenzuteilung während des ersten Lebensjahres begleitet und von uns zu drei verschiedenen Zeitpunkten untersucht.
Das geschieht ***a)*** *<u>nach Austritt aus dem Kinderspital Zürich</u>* (im Alter von 3-6 Monaten) und
b) *<u>drei bis vier Monate</u>* <u>später</u> (im Alter von 6-9 Monaten) im Kinderspital. Wir untersuchen die motorische Entwicklung Ihres Säuglings mittels Videoaufnahme, spielerisch in Rückenlage, Bauchlage und im Sitzen und dauert 15 Minuten.
c) Am *<u>Ende der Studie</u>* (im Alter von 12 Monaten Ihres Säuglings) findet am Kinderspital Zürich routinemässig eine entwicklungsneurologische Untersuchung statt. In einer spielerischen Testsituation untersuchen wir die motorische, kognitive und sprachliche Entwicklung. Im Anschluss an diese Untersuchung erhalten Sie eine Auswertung des Entwicklungsstandes Ihres Kindes. Keine dieser Untersuchungen wird der Krankenversicherung in Rechnung gestellt.
An allen drei Zeitpunkten erfassen wir zusätzlich mit Hilfe von Fragebögen wie es Ihnen und Ihrem Kind während der Studienzeit geht.

Wird Ihr Kind in die Interventionsgruppe eingeteilt, werden Sie und Ihr Säugling, zusätzlich zu den oben beschriebenen Testzeitpunkten, während drei Monate von einer Physiotherapeutin begleitet. Diese wird mit Ihnen gemeinsam Ihr Kind in seiner motorischen Entwicklung im Alltag unterstützen. Die Begleitung findet abwechselnd bei Ihnen zu Hause, am Kinderspital Zürich und über Videokonferenzen mittels eines sicheren Onlineprogramms statt. Zudem findet bei Bedarf eine telefonische Beratung statt. Die Therapiestunden werden auf Video aufgezeichnet und bezüglich der Interaktion zwischen der Therapeutin, Ihnen und Ihrem Kind ausgewertet.

5. Nutzen für die Teilnehmenden

Dank Ihrer Teilnahme helfen Sie uns zu untersuchen, ob eine motorische Frühförderung bei Kindern mit angeborenen Herzfehlern deren Entwicklung unterstützt. Dadurch gewinnen wir neuen Erkenntnisse über neue therapeutische Massnahmen. Als Gegenleistung begleiten wir Sie und Ihr Kind nach Austritt aus dem Kinderspital und begleiten die Entwicklung Ihres Kindes nach neuersten Erkenntnissen. Selbstverständlich informieren wir Sie über die Resultate der Untersuchung ihres Kindes als auch die Resultate der Studie.

6. Rechte der Teilnehmenden

Die Teilnahme Ihres Kindes an der Studie ist freiwillig. Wenn Sie auf die Teilnahme an dieser Studie verzichten, hat Ihr Kind keine Nachteile für seine weitere medizinische Betreuung. Das gleiche gilt, wenn Sie Ihre gegebene Einwilligung zu einem späteren Zeitpunkt widerrufen. Diese Möglichkeit haben Sie jederzeit.

7. Pflichten der Teilnehmenden

Als Studienteilnehmende sind Sie verpflichtet:
- den Anweisungen der Prüfperson zu folgen und sich an den Studienplan zu halten
- die Prüfperson genau über den Verlauf der Erkrankung zu informieren
- die Prüfperson über die gleichzeitige Behandlung Ihres Kindes bei einer anderen Therapeutin/Therapeuten, einer anderen Ärztin/ Arzt und über die Einnahme von Arzneimitteln zu informieren. Zu den Arzneimitteln gehören auch alle selbstgekauften, ohne ärztliches Rezept erhältlichen und/oder alternativmedizinischen Präparate (Kräuter, Pflanzen, homöopathische, asiatische Heilmittel, Speziallebensmittel und Vitamine).

8. Risiken und Unannehmlichkeiten

Die Unterstützung der motorischen Entwicklung Ihres Kindes und die Untersuchung dieser sowie die Erhebung Ihrer Befindlichkeit und die Ihres Kindes birgt keine Risiken und Unannehmlichkeiten.
Nur ein bis vier Besuche Ihres Kindes am Kinderspital Zürich (je nach Gruppeneinteilung) sind mit einem zeitlichen Aufwand und der Anreise verbunden.

9. Neue Erkenntnisse

Die Prüfperson informiert Sie über alle neuen Erkenntnisse, die den Nutzen und somit Ihre Einwilligung zur Teilnahme Ihres Kindes an der Studie beeinflussen können, schriftlich. Bei neuen Ergebnissen während der Studie werden Sie informiert. Falls Sie dies nicht möchten, informieren Sie bitte die Prüfperson.

10. Vertraulichkeit der Daten

Persönliche studienbedingte erfasste Daten von Ihnen und Ihrem Kind werden nach Erhebung verschlüsselt. Sie sind dem Studienteam zur wissenschaftlichen Auswertung zugängig. Verschlüsselung bedeutet, dass alle Bezugsdaten, die Ihr Kind identifizieren könnten (Name, Geburtsdatum) gelöscht und durch einen Schlüssel (Code) ersetzt werden. Diejenigen Personen, die den Schlüssel nicht kennen, können daher keine Rückschlüsse auf Ihr Kind ziehen. Die Schlüssel-Liste bleibt immer im Kinderspital Zürich. Alle Personen, die Einsicht in die Projektdaten haben, unterliegen der Schweigepflicht.
Möglicherweise wird dieses Studienprojekt durch die zuständige Ethikkommission oder durch die Institution, die das Projekt veranlasst hat, vor Ort überprüft. Der Projektleiter muss für solche Kontrollen die persönlichen und medizinischen Daten offenlegen. Während der gesamten Studie und bei den erwähnten

Kontrollen wird die Vertraulichkeit strikt gewahrt. Ihr Name und der Ihres Kindes werden in keiner Weise in Rapporten oder Publikationen, die aus der Studie hervorgehen, veröffentlicht. Die Daten werden für andere Forschungsprojekte weiterverwendet, wenn ein separates Einverständnis dafür erteilt wird (siehe Anhang).

11. Rücktritt

Einen allfälligen Widerruf Ihrer Einwilligung bzw. den Rücktritt von der Studie müssen Sie nicht begründen. Im Falle eines Widerrufes werden die bis zu diesem Zeitpunkt erhobenen Daten weiterverwendet. Nach der Auswertung werden die Daten Ihres Kindes vollständig anonymisiert, d.h. die Schlüsselzuordnung wird vernichtet, sodass niemand erfahren kann, dass die Daten von Ihrem Kind stammen.

12. Kosten

Die in dieser Patienten-Information erwähnten studienspezifischen entwicklungsunterstützenden Behandlungen und Untersuchungen sind kostenlos und werde nicht der Krankenversicherung verrechnet. Reisespesen werden Ihnen am Ende der Studie vergütet.

13. Entschädigung für die Teilnehmenden

Für die Teilnahme an diesem Forschungsprojekt erhalten Sie keine Entschädigung. Ihr Säugling erhält als Dankeschön für die Teilnahme aber ein kleines Geschenk in Form eines Kinderbuches oder Spielzeug.

14. Haftung

Die Haftpflichtversicherung des Kinderspitals Zürich kommt für allfällige Schäden auf.

15. Finanzierung

Dieses Projekt wird von der Anna Müller Grocholski-Stiftung finanziell unterstützt.

16. Kontaktpersonen

Bei Unklarheiten, Notfällen, unerwarteten oder unerwünschten Ereignissen, die während der Studie oder nach deren Abschluss auftreten, können sie sich jederzeit an die untenstehenden Kontaktpersonen wenden.

Frau
Prof. Dr. med. Bea Latal
Abteilung Entwicklungspädiatrie
Steinwiesstrasse 75
8032 Zürich
Tel. +41 44 266 77 51
E-mail: bea.latal@kispi.uzh.ch

Frau
Elena Mitteregger, MScPT
Abteilung Entwicklungspädiatrie
Steinwiesstrasse 75
8032 Zürich
Tel. +41 44 266 76 90
Email: elena.mitteregger@kispi.uzh.ch

Schriftliche Einverständniserklärung der Eltern zur Teilnahme Ihres Kindes an einer Studie

- Bitte lesen Sie dieses Formular sorgfältig durch.
- Bitte fragen Sie, wenn Sie etwas nicht verstehen oder wissen möchten.

**Nummer der Studie:**	BASEC 2019-01787
**Titel der Studie:**	Frühintervention bei Säuglingen mit angeborenen Herzfehlern
**Sponsorin**	Prof. Dr. med. Bea Latal, MPH, Abteilung Entwicklungspädiatrie
**Investigator:**	Elena Mitteregger, MScPT, Abteilung Entwicklungspädiatrie
	Kinderspital Zürich, Steinwiesstrasse 75, 8032 Zürich
**Ort der Studie:**	Kinderspital Zürich
**Prüfpersonen:**	Prof. Dr. med. Latal, Bea, MPH und Elena Mitteregger, MScPT
**Patient/ Patientin**	☐ männlich ☐ weiblich
Name und Vorname:	
Geburtsdatum:	
**Eltern**	
Name und Vorname:	

- Wir wurden von der unterzeichnenden Prüfperson mündlich und schriftlich über die Ziele, den Ablauf des Forschungsprojektes, über die zu erwartenden Wirkungen, über mögliche Vor- und Nachteile sowie über eventuelle Risiken informiert.
- Wir haben die zur oben genannten Studie abgegebene schriftliche Elterninformation (Version 1.1) gelesen und verstanden. Unsere Fragen im Zusammenhang mit der Teilnahme an dieser Studie sind uns zufriedenstellend beantwortet worden. Wir können die schriftliche Elterninformation behalten und erhalten eine Kopie unserer schriftlichen Einverständniserklärung.
- Wir hatten genügend Zeit, um unsere Entscheidung zu treffen.
- Wir sind darüber informiert worden, dass eine Versicherung Schäden deckt, falls solche im Rahmen der Studie auftreten sollten.
- Wir sind einverstanden, dass die Kinderärztin/ der Kinderarzt über die Studienteilnahme informiert wird.
- Wir wissen, dass unsere persönlichen Daten und die unseres Kindes nur in verschlüsselter Form an aussenstehende Institutionen im Rahmen dieses Forschungsprojektes weitergegeben werden. Wir sind einverstanden, dass die zuständigen Fachleute der Kantonalen Ethikkommission zu Prüf- und Kontrollzwecken in unsere Originaldaten Einsicht nehmen dürfen, jedoch unter strikter Einhaltung der Vertraulichkeit.
- Wir nehmen an dieser Studie freiwillig teil. Wir können jederzeit und ohne Angabe von Gründen unsere Zustimmung zur Teilnahme widerrufen, ohne dass unserem Kind deswegen Nachteile bei der weiteren medizinischen Betreuung entstehen.
- Wir sind uns bewusst, dass während der Studie die in der Elterninformation genannten Anforderungen und Einschränkungen einzuhalten sind.

- Im Interesse der Gesundheit unseres Kindes kann der Prüfperson unser Kind jederzeit von der Studie ausschliessen. Zudem orientieren wir die Prüfperson über die gleichzeitige Behandlung unseres Kindes bei einer anderen Therapeutin/ Therapeuten, bei einer anderen Ärztin/ Arzt sowie über die Einnahme von Medikamenten (verordnete oder selbständig gekaufte).

Ort, Datum	Unterschrift der Eltern des Kindes

Bestätigung der Prüfperson: Hiermit bestätige ich, dass ich den Eltern des Patienten/ der Patientin Wesen, Bedeutung und Tragweite der Studie erläutert habe. Ich versichere, alle im Zusammenhang mit dieser Studie stehenden Verpflichtungen zu erfüllen. Sollte ich zu irgendeinem Zeitpunkt während der Durchführung der Studie von Aspekten erfahren, welche die Bereitschaft der Eltern des Patienten/ der Patientin zur Teilnahme an der Studie beeinflussen könnten, werde ich sie umgehend darüber informieren.

Ort, Datum	Unterschrift der Prüfperson

Einwilligungserklärung für die Weiterverwendung Ihrer Daten für die Forschung

Name und Vorname des/r Probanden/in: ………………………

Geburtsdatum: ………………

Die im Kinderspital zuständigen, genau bezeichneten Personen verschlüsseln die erhobenen Daten sowie die medizinischen und personenbezogenen Daten, bevor sie für einzelne Forschungsprojekte verwendet werden. Das bedeutet, dass Personen, die an Forschungsprojekten nach Abschluss der ursprünglichen, oben genannten Studie beteiligt sind, nicht wissen, dass die Daten von Ihrem Kind stammen.

Die Daten meines Kindes dürfen nur in verschlüsselter Form an andere Institutionen im In- und Ausland für die Forschung weitergegeben werden. Zudem muss gewährleistet sein, dass dieselben Anforderungen an den Datenschutz garantiert sind.
Jedes Forschungsprojekt muss von der zuständigen Ethikkommission bewilligt werden.

Ich bestätige, dass

- ich die zu dieser Einwilligungserklärung gehörende Information erhalten habe.
- ich hinreichend über das Vorgehen, das sich aus meiner Einwilligung ergeben hat, informiert wurde.
- ich hinreichend über die Weiterverwendung von Daten für die Forschung informiert wurde.
- ich die Möglichkeit hatte, Fragen zu stellen und diese zu meiner Zufriedenheit beantwortet wurden.
- meine Einwilligung freiwillig ist und mir insbesondere keine Vor- oder Nachteile entstehen, wie ich mich auch entscheide.
- ich weiss, dass ich diese Einwilligung jederzeit widerrufen kann, ohne Angabe von Gründen.

Für Fragen stehen Ihnen zur Verfügung:

Frau
Prof. Dr. med. Bea Latal
Abteilung Entwicklungspädiatrie
Steinwiesstrasse 75
8032 Zürich
Tel. +41 44 266 77 51
E-mail: bea.latal@kispi.uzh.ch

Frau
Elena Mitteregger, MScPT
Abteilung Entwicklungspädiatrie
Steinwiesstrasse 75
8032 Zürich
Tel. +41 44 266 76 90
Email: elena.mitteregger@kispi.uzh.ch

Ort, Datum	Unterschrift der Eltern des Kindes
