# Supplementary material for: A family-tailored early motor intervention (EMI-Heart) for infants with complex congenital heart disease: study protocol for a feasibility RCT
Source: Pilot Feasibility Stud. 2022 Dec 23;8:263. doi: 10.1186/s40814-022-01220-y (PMC9784271; doi:10.1186/s40814-022-01220-y)
Supplement: Supplementary file 3 — Additional file 3. Study specific monitoring plan. [file 40814_2022_1220_MOESM3_ESM.pdf]

Part I: Study-specific Monitoring Plan

1. Study Details

**Study Title**	Family centred intervention for infants with CHD
Product Name, if available	EMI Heart
Study Protocol Version and Date	EMI Heart Protocol_version 1.1 (16.04.2021)
Sponsor Study Code	EMI Heart, 2019.INV-022 (FZK Nummer)

**Sponsor**	
Contact Person<br>(Title, First name, Name, Function)	Prof. Dr. med Bea Latal, MPH<br>Head of Child Development Center
Address	University Children's Hospital Zürich<br>Steinwiesstrasse 75, 8032 Zürich
Tel., e-mail	bea.latal@kispi.uzh.ch<br>044 266 7751

**Contact Person for Monitoring**	
Contact Person<br>(Title, First name, Name, Function)	Elena Mitteregger, MScPT<br>Principal Investigator
Address	University Children's Hospital Zürich<br>Steinwiesstrasse 75, 8032 Zürich
Tel., e-mail	elena.mitteregger@kispi.uzh.ch<br>044 266 7690

2. Approval Details

Clinical Monitoring Plan Approval	
Version/ Date of Monitoring Plan	*version 1.1*
Release Date (*dd-mmm-yyyy*)	
Author of Monitoring Plan	Elena Mitteregger, MScPT
Date and Author Signature	16.04.2021
Reviewer of Monitoring Plan	Forschung Zentrum für das Kind (FZK)
Date and Reviewer Signature	Siehe beiliegende FZK Monitorvereinbarung von 15.03.2021<br>FZK Monitoringvereinbarung EMI Heart Studie.pdf

Sponsor	
Sponsor Contact Name:	Prof. Dr. med. Bea Latal, MPH
Sponsor Contact Function:	Head of Child Development Center
Sponsor Contact Signature:	
Date	16.04.2021

3. General Information

The Monitoring Plan consists of two parts. General monitoring terms are listed in part II (Attachment to Monitoring Plan). In part I -in the actual Monitoring Plan- study specific conditions are regulated. Study specific agreements overrule standard statements of the attachment.

This Monitoring Plan is based on the clinical study-specific requirements and the agreed upon commitments between

Prof. Dr. med. Bea Latal, MPH, Head of Child Development Center

and

Forschungszentrum für das Kind (FZK) of the University Children's Hospital Zurich not included into the study will fulfil the monitoring duties.

If agreed commitments change after study start, the contract will be amended and if necessary, a new version of Monitoring Plan will be issued. Service provider will act as Monitor on behalf of Sponsor for this study. Monitor (administrative) and Principal Investigator (medical questions) are the first line of contact for study site.

4. Study Design

4.1 Study sites

University Children's Hospital Zurich, Switzerland, planned patients involved approximately 15-20.

This Monitoring Plan is valid for the above-mentioned site.

4.2 Summary

Children considered at high risk of neurodevelopmental disorders include children with severe congenital heart disease. Despite this knowledge there is no standard early motor intervention available in Switzerland for this patient group. Thus, we want to conduct a feasibility pilot study investigating if early family centered intervention promotes infant's motor development in children with congenital heart disease.

- **Endpoints***
  - Infant Motor Profile
  - The Alberta Infant Motor Scale
  - General Movements
  - Hammersmith Infant Neurological Examination
  - The Pediatric Quality of Life Inventory
  - Eltern Belastungs Inventar
  - Brief Symptom Inventory 18
  - Quality of life short form 36
  - Bayley Scale for Infant and Toddlers Development III
  - General Movements
  - Parental Overprotection Measure
  - Family Empowerment Scale

- **Inclusion criteria***
  - Infants born with severe cyanotic and acyanotic CHD (e.g. tetralogy of Fallot, pulmonary atresia, ventricular septal defect, atrioventricular septal defect, transposition of the great arteries, double right outlet right ventricle) who undergo open-heart surgery once before the first 3-6 months of their life
  - Parents able to comprehend the patient information linguistically and cognitively
  - Informed consent of parents as documented by signature

- **Exclusion criteria***
  - Infants with hypoplastic left heart syndrome as they need to undergo several open-heart surgeries within the first period of their life
  - Infants with trisomy 21 and other syndromes like 22q11 microdeletion syndrome, CHARGE syndrome that are often associated with CHD. These infants often show additional cognitive impairments that might confound the effect of our intervention.

* Relevant is the last approved version of the protocol or protocol amendment

4.3 Study Timeline

Monitor training (study specific) by Sponsor ☐ no ☒ yes	
Initiation Visit	Before first enrolment, approx. April 2021
First enrolment	Approx. May 2021
Last patient last visit (including follow up period if applicable)	Approx. April 2023
Query resolution complete	by monitor
Database lock	end of study
Close-out visit (after database lock)	Appox. Dec. 2024

4.4 Extend of Monitoring based on risk-adapted approach

Details and amount of monitoring are in general defined by the risk category according to the Human Research Act and confirmed by the SCTO-"Monitoring Risk Analysis". Further criteria (such as experience of a site, use of a validated database, function as the coordinating site) could increase or decrease the extend of monitoring for a study.

HRA risk category	A
Risk-adapted Monitoring Class according to SCTO Monitoring Risk Analysis	Low risk
Risk score based on evaluation of further criteria	0-1= risk decrease

Recommended extend of monitoring Based on above documented risk analysis	1x site initiation visit<br>1x after enrolment of two participants<br>1x after 10 participants<br>(2x during the study)<br>1x close-out visit

4.5 Monitoring Timeline

Study Period	Schedules	Hours on site/ in-house
Site initiation visit	Prestudy visit	2 hours on site and for preparation at service provider
Monitoring visit 1	After 2 included participants	2 hours on site and for preparation at service provider
Monitoring visit 2	After 10 included participants	2 hours on site and for preparation at service provider
Close-out visit 3	After last included participant	2 hours on site and for preparation at service provider
Regular communication	Updates on a regular basis	In house with FZK

4.6 Scope of Monitoring

Action	Extent (%)	Comments
Patient information and in-formed consent	100%	
Inclusion/exclusion	30%	
Primary Endpoint	20%	
Secondary Endpoint	20%	
Safety (AE/SAE)	100%	
TMF/ISF	100%	
Sample storage & logistics	20%	
Data set	100%	
Delegation and Training Log	100%	
Approval from Research Ethics Committee	100%	
Original Protocol/Protocol Signature Page	100%	
Study specific SOPs	30%	
Trial Master File	100%	
Source verification against clinical file	30%	

The Monitor must notify line management and Sponsor if additional time or resources are necessary.

5. Documentation and Communication of Protocol Deviations

A protocol violation or deviation is any failure to comply with the protocol. If applicable the following protocol violations/deviations (as well as the necessary corrective actions) need to be reported in the Monitoring Report and discussed with Investigator.
In case of severe violations line management and Sponsor must be informed immediately during the same visit:

- Informed consent process not adequately performed
- Violation of Inclusion/Exclusion criteria
- Serious Adverse Event & Adverse Event reporting requirements not followed
- Subject follow ups and visits not performed correctly as described in the protocol
- Required tests and sampling procedures incorrectly or not performed
- Any GCP non-compliance

All deviations/violations must be reported by monitor in follow-up letters & monitoring visit reports. The Monitor must confirm that the site has properly notified Sponsor and Independent Ethics Committee (IEC).

6. Safety Reporting (if applicable)

6.1 Adverse Event (AE)

Adverse Events (AE) must be documented according to the valid version of the GCP Basisprozess. If an AE is continuing, the Monitor will follow it up at the next visit and each subsequent visit, as applicable, until the AE is resolved.

6.2 Serious Adverse Event (SAE)

Serious Adverse Events must be reported immediately (within 24 hours after detection) to Sponsor. Independent Ethic Committee and Competent Authority have to be informed according to the valid version of the GCP Basisprozess “Meldung von unerwünschten Ereignissen (AEs, SAEs und SUSARs)”.

Source document verification of the information captured on the SAE form has to be performed during the next monitoring visit at the site.

If the Monitor identifies a SAE during the monitoring visit, which has not been previously reported, the Monitor will request the site to complete a SAE form and to inform Sponsor and Independent Ethics Committee as per legally defined timelines.

Investigator and Sponsor have to be informed by the monitor accordingly and have to be advised that they must comply with all reporting requirements as described in the valid version of the GCP Basisprozess “Meldung von unerwünschten Ereignissen (AEs, SAEs und SUSARs)”.

7. Audit/ Inspection

If any of the parties involved in the study becomes aware that an audit/ inspection by a competent Authority or others is planned, all other parties have to be informed immediately in order to prepare for the visit.

8. Contact Details

8.1 Study Sites

Site Name & No.	University Children's Hospital Zurich
Contact Person	Prof. Dr. med. Bea Latal, MPH
Function	Sponsor, Head of Child Development Center
Address	Steinwiesstrasse 75, 8032 Zürich
Tel., Fax, e-mail	bea.latal@kispi.uzh.ch<br>044 266 7751

Data Management

Name	Elena Mitteregger, MScPT
Function	PI, PhD student
Address	Steinwiesstrasse 75, 8032 Zürich
Tel., Fax, e-mail	elena.mitteregger@kispi.uzh.ch<br>044 266 7690

Part II: Attachment to Monitoring Plan

1. Introduction

1.1 Monitoring Training

Compliance with protocol requirements is important to ensure ethical conduct of clinical trial, the scientific validity, and accuracy and completeness of data produced during the clinical study. Monitoring helps to ensure compliance.

In particular, the monitor(s) have to be trained in:

- Any general monitoring requirements, e.g. protocol, Case Report Form (CRF), Source Data Verification (SDV)
- GCP Basisprozesse
- Regulations and Guidelines in the actual valid version
  - ~ International Conference on Harmonization – Good Clinical Practice
  - ~ Humanforschungsgesetz (HFG)
  - ~ Verordnung über klinische Versuche KlinV
  - ~ Medizinprodukteverordnung (MepV)

As employer of the initially assigned monitor the service provider will be responsible for study specific training/ handover from assigned Monitor to any Monitor later joining the project.

1.2 Study Specific Training

Sponsor will be responsible for study specific training. Training has to be documented in a signed study training log which will be filed in the Trial Master File (TMF). A copy of all training related presentations and further information has to be filed as well.

The training should cover at least:

- Medical background and endpoint training
- Study design
- Study timelines
- (e)Case Report Form (CRF) composition and completion
- Query-processing/ Data base lock

Study specific training is basically meant for the site. In addition, Monitor should be trained by the sponsor with respect to monitoring issues.

2. Course of Events

2.1 Correspondence

All relevant study related correspondence (between sponsor/ site/ service provider and external parties) by letter, e-mail or telephone should be documented and filed into TMF.

2.2 Confirmation of Visit

All visits will be preceded by a confirmation letter including agenda (date, time, place of visit, requested documents and source data attendees, etc.) sent to the investigator (and other staff, if applicable).

It is acceptable to fax or e-mail the confirmation letter.

2.3 Initiation Visit

If determined per contract, the site will be initiated. According to GCP the site initiation visit has to be performed after study approval but prior to subject enrolment.

During the initiation visit the Monitor can be supported by other team members that are delegated by the sponsor.

The following topics are taken into account during study initiation:

- Set up **personal contacts** with site staff
- **Train** whole study team focussed on study specific procedures, safety and regulatory issues, responsibilities, timelines, study procedures, monitoring procedures, administrations, protocol deviations and consequences
- Inform about the requirement to document critical findings
- Discuss **GCP** and remind on regulatory requirements
- Discuss anticipated **schedule of patient visits** and which activities will occur at each visit (routine work/ additional work for the study)
- If applicable, review and explain **record keeping** per site and subject
- Review and explain **TMF/ ISF completeness** (e.g. IB, signed clinical protocol, etc.) and availability of all required regulatory documents with responsible site personnel. Monitor will provide instructions to site personnel on organization and maintenance of documents in TMF
- Ensure authorisation of study staff by investigator (log of functions and responsibilities)
- Instruct site personnel in **Informed Consent process** and requirements
- Review and explain **AE/ SAE reporting** procedures and documentation
- Remind Investigator's **reporting responsibilities** to Independent Ethics Committee/ Authorities/Sponsor (safety, annual report, closure of site)
- Review **CRF and completion procedures**
- Review source documentation and **Source Data Verification** (SDV)
- Discuss **Data Clarification Form** (DCF) procedure and Data management process
- Ensure **Site Visit Log** is signed

Once the site initiation visit is completed and all necessary material is available, the site will be authorized by the sponsor to start enrolment ("green light"-process).

After the visit Monitor will write a visit report and a follow-up letter/e-mail.

2.4 Interim Monitoring Visit

Monitoring visits will be conducted in order to help the site following all study requirements as specified in the protocol and in ICH/GCP and to assist with anything needed to ensure the rights, wellbeing and safety of the subjects as well as to establish good data quality. Additional persons (co-monitor, designee of sponsor, EC or authority) may support the Monitor during the visit.

During the Monitoring Visit the Monitor will:

- Check whether **changes in responsibilities of site personnel** have occurred
- If necessary, train new site personnel on the protocol and study procedures
- Confirm that **site facility** remains adequate for performing the study
- Check and confirm that site is compliant with **patient assessments** as per protocol
- Check and confirm **adherence to protocol** and document protocol violations/deviations in a Note to File (NTF). Monitor must communicate violations/deviations to the Investigator/ sponsor
- Review **enrolment** including number of subjects screened, completed and withdrawn. If necessary, discuss options to increase patient recruitment.
- Check that there is a correctly signed **Informed Consent Form** (ICF) for each patient in the approved version. Remind site to hand over a copy of the signed ICF to patients.
- Check that withdrawals are appropriately documented including the reasons for **withdrawal**

- Verify that subjects meet **inclusion and exclusion criteria** by comparing with health records as defined in the scope of monitoring (Section 4.7; Part I)
- Review **AE/ SAE/ pregnancies** documentation and reporting and note any serious and/or unexpected adverse effects, including all follow-up actions taken. Ensure that for each SAE a complete SAE report has been submitted to the responsible persons/ authorities as specified in the GCP Basisprozess "Meldung von unerwünschten Ereignissen (AEs, SAEs und SUSARs)".
- Perform **source data verification** (SDV) on CRF data including queries in order to assess subject compliance, safety, and integrity of data
- Check **query resolution** by site and verify source data for query resolution
- **Collect** completed and verified **CRFs and DCFs**
- Review TMF **completeness** as defined in the scope of monitoring (Section 4.7; Part I)
- Identify **action items** for site personnel and/or Monitor
- **Discuss findings with investigator/ site staff**
- Record visit in **site visit log**

All ongoing issues from the last Monitoring visit should be reviewed and resolved, if possible.

After the visit Monitor will write a visit report and a follow-up letter.

2.5 Source Data Verification (SDV)

The Monitor verifies CRF data by comparing with corresponding source data (medical charts -paper or online- data or source documents including e.g.: laboratory reports, print outs of electronic data, etc.).

The Monitor:

- Ensures that discrepancies are documented in the appropriate comments section of the monitoring visit report.
  Same applies if a subject has completed participation, but site has not completed the CRF: also, in this case the discrepancy has to be documented and the site is instructed to complete the CRF.
- Confirms that AEs, concomitant medications and concurrent illnesses have been entered into the appropriate sections and that cross-references to AEs and concomitant medications are correct.
- Confirms that missed patient visits, tests or examinations are adequately documented in CRF.
- If agreed with sponsor, paper CRFs respectively print-outs of eCRFs have to be collected. Collection and transmission of paper CRFs have to be documented, e.g. in a document transmission form.
  Completed electronic CRF are frozen after validation. Print-outs are stored at site.
- Performs administrative review regarding internal logic, consistency and completeness of data.

Screening failure subjects must be listed in a log. For these subjects only ICF and reason for failure will be verified/ monitored against source data (In-/ Exclusion Criteria).

All unused paper-CRFs will be returned to Sponsor or destroyed at site (decision of Sponsor). Any destruction has to be documented; documentation will be filed in TMF.

2.6 Data Clarification Form (DCF)

DCFs (queries) that are distributed to site must be resolved and returned to data management (DM) within the requested timeline. In case of paper CRFs copies of the DCFs will be filed at site together with the corresponding CRFs, originals will be forwarded to DM.

The DCFs must be verified against source data and signed by PI or designee. (Signatures only if paper is used).

If any DCF is sent to DM without being previously reviewed, the Monitor will monitor the data during the next monitoring visit.

2.7 Close-out Visit

Close-out Visit will be conducted to close the study at a site after data base lock.

During the Close-out Visit Monitor will:

- Ensure **completeness of TMF** and update file if necessary
- Ensure that **all forms and logs are completed correctly** and signed by PI
- Verify that **subject identification** is complete and filed anonymously in TMF. The original is filed in ISF. For investigator initiated studies TMF and ISF may be combined as one file and maintained at the site, but all non-anonymous documents must be filed in a separate chapter within the file.
- Ensure that all **study related documents** are on file and remind investigator of archiving requirements and retention times (e.g. ISF, Patient Data, Diaries, copies of CRF).
- **Inform investigator** about:
    - ~ Responsibility for ongoing AE and SAE
    - ~ Obligation to inform ethic committee in writing about closure of site
    - ~ Obligation to inform Competent Authority (if involved) in writing about closure of site and end of study (Sponsor-Investigator) including final SAE information
    - ~ Possibility of audits or inspections by authorities, ethic committees or sponsor

After the visit Monitor will write a visit report and a follow-up letter.

2.8 Visit Report

Following each Site Visit (Initiation Visit, Interim Monitoring Visit, Close out Visit), the Monitor writes a Visit Report. All Study Visit Reports issued by the Monitor will be reviewed internally before submitting them to Sponsor for final approval and signature.

The Visit Report is submitted to Sponsor within 15 business days, internal review included.

2.9 Follow-up Letter

After each visit the Monitor also writes a follow-up letter to the site that includes comments to the following items:

- Review of all study issues discussed with Investigator/ site staff
- Scope of monitoring
- Protocol and/or regulatory deviations identified
- Action items that need to be addressed by Principal Investigator (all action items must be directed to the PI, however he/ she can delegate to other study staff as needed)

The follow-up letter must be forwarded to the investigator within 5 business days after the monitoring visit. It is acceptable to fax or e-mail the follow up letter to the site.

2.10 Filing of Documents

	Trial Master File	Investigator Site File
Monitoring Plan	Original	----
Confirmation letters	Copy	Original
Pre-study visit	Original	----
Initiation Visit Report	Original	Copy
Interim Monitoring Visit Report	Original	----
Close-out Visit Report	Original	Copy
Follow-up letters	Copy	Original
Correspondence	Original/ Copy	Original/ Copy
