# Supplementary material for: A family-tailored early motor intervention (EMI-Heart) for infants with complex congenital heart disease: study protocol for a feasibility RCT
Source: Pilot Feasibility Stud. 2022 Dec 23;8:263. doi: 10.1186/s40814-022-01220-y (PMC9784271; doi:10.1186/s40814-022-01220-y)
Supplement: Supplementary file 4 — Additional file 4. Trial Register Data Set. [file 40814_2022_1220_MOESM4_ESM.pdf]

Trial Registration Data Set

Data category	Information
Primary registry and trial identifying number	Clinicaltrials.gov (NCTT04666857)
Date of registration in primary registry	14.12.2020
Source(s) of monetary or material support	grant from the Anna Mueller Grocholski Foundation
Trial sponsor	Prof. Dr. med. Beatrice Latal; bea.latal@kispi.uzh.ch<br>Child Development Center, University Children's Hospital Zurich, Switzerland
Principal investigator	Elena Mitteregger, MScPT; elena.mitteregger@kispi.uzh.ch<br>Child Development Center, University Children's Hospital Zurich, Switzerland
Contact for public & scientific queries	Elena Mitteregger, MScPT; elena.mitteregger@kispi.uzh.ch
Scientific title	A family-tailored early motor intervention (EMI-Heart) for infants with complex congenital heart disease:<br>study protocol for a feasibility RCT
Country of recruitment	Switzerland
Health condition(s) or problem(s) studied	Motor development of infants with complex congenital heart disease
Intervention(s)	Intervention group: early motor intervention<br>Control group: standard of care
Key inclusion and exclusion criteria	Inclusion criteria:<br>(1) infants with CHD, (2) infants born ≥ 37 weeks gestational age, (3) infants aged 3–5 months at start of intervention, who underwent open heart surgery with cardiopulmonary bypass once within the first 5 months of life, (4) infants discharged home before the age of 6 months, (5) informed consent of infants' parents documented by signature, and (6) families living within an hour's journey from the Children's Hospital.<br>Exclusion criteria:<br>(1) infants with univentricular heart defects, (2) infants with syndromes that are often associated with CHD and worse neurodevelopmental outcomes, (3) large cerebral and clinically manifest lesions, (4) infants whose parents have an inadequate understanding of the German language and are thus unable to comprehend the patient information
Study type	Interventional study<br>Allocation: randomized controlled<br>Intervention model: parallel assignment<br>Masking: single blinded (outcomes assessors)<br>Primary purpose: feasibility<br>Phase I
Date of first enrolment	June 2021
Target sample size	20
Recruitment status	Recruiting
Primary Outcome(s)	Feasibility
Key secondary outcomes	motor development and families' well-being

Protocol version: 01.02.22, Version 1.3
